# Knowledge mapping of immunotherapy in cervical carcinoma: a bibliometric analysis (2000-2023)

**DOI:** 10.3389/fimmu.2023.1328103

**Published:** 2024-01-09

**Authors:** Ling Song, Xinmei Liang, Min Zhu, Qiang Su, Fengzhou Li

**Affiliations:** ^1^ Clinical Laboratory Medicine, Binhai New Area Hospital of Traditional Chinese Medicine, Fourth Teaching Hospital, Tianjin University of Traditional Chinese Medicine, Tianjin, China; ^2^ Clinical Laboratory Medicine, Beijin Shijitan Hospital, Capital Medical University, Beijing, China; ^3^ The First Affiliated Hospital, Dalian Medical University, Dalian, Liaoning, China

**Keywords:** cervical carcinoma, immunotherapy, VOSviewers, CiteSpace, bibliometric analysis

## Abstract

**Background:**

Cervical carcinoma is a type of malignant tumor that primarily develops in the cervix, the lower part of the uterus. In recent years, Despite the considerable progress made in immunotherapy research for cervical carcinoma, an important aspect has been largely overlooked - the absence of a comprehensive bibliometric analysis in this field. By employing bibliometric techniques, this study aims to fill this gap and provide a comprehensive overview of the knowledge structure and research hotspots within the realm of immunotherapy in cervical carcinoma.

**Method:**

A comprehensive search was conducted on the web of science core collection(WoSCC) database to identify publications related to immunotherapy specifically for the treatment of cervical carcinoma. The search spanned the period from the year 2000 to 2023. Several analytical tools were employed. These included VOSviewers, CiteSpace, and the R package “bibliometrix”.

**Results:**

A total of 654 research articles from 66 different countries have been included in the analysis. The United States and China have emerged as the leading countries in publishing research on immunotherapy in cervical carcinoma. Leiden University and Memorial Sloan-Kettering Cancer Center from the Netherlands and the United States respectively have a close cooperation. Fudan University from China and the German Cancer Research Center are also among the key institutions leading research in this area. Frontiers in Oncology has emerged as the most popular and widely recognized publication in the field of immunotherapy in cervical carcinoma. Journal of Clinical Oncology is frequently cited by researchers in this area. Van Der Burg, Sjoerd H has published the highest number of papers. Tewari, Krishnansu S has been the most co-cited author. Keywords such as PD-L1, chemotherapy, and immune checkpoint inhibitors have gained significant attention in recent years.

**Conclusion:**

This is the first bibliometric study that comprehensively summarizes the research trends and developments of immunotherapy in cervical carcinoma. This groundbreaking study not only summarizes the current research trends and developments in immunotherapy for cervical carcinoma but also provides a reference for scholars studying the treatment of cervical cancer.

## Introduction

Cervical cancer is the fourth most common cancer among women worldwide and is caused by a persistent infection with high-risk strains of the human papilloma virus (HPV), with HPV16 and/or HPV18 being the most frequent culprits (1, 2). According to the International Agency for Research on Cancer, cervical cancer is the leading cause of death among women globally, particularly in developing countries (3, 4). Currently, although significant progress has been made in preventing cervical cancer through HPV vaccines and cancer screening, the outlook for patients in the advanced stage of the disease remains grim. Cervical cancer becomes resistant to chemotherapy in its advanced stages, making it difficult to achieve long-term palliative care or even a cure. Some researcher efforts to develop therapeutic vaccines targeting the HPV oncoproteins, these attempts have proven unsuccessful (5). With the continuous research on immunotherapy, there is growing evidence from research that it can lead to the regression of cervical cancer.

Immunotherapy is a revolutionary treatment approach that utilizes the body’s own immune system to specifically target and destroy cancer cells (6, 7). Unlike traditional treatments such as chemotherapy or surgery, which directly kill or remove cancer cells, immunotherapy focuses on boosting the body’s natural defense mechanisms to fight against cancer. Chemotherapies and other agents that directly kill cancer cells have a higher chance of damaging healthy cells in the process (8). Immunotherapy focuses specifically on enhancing the immune response against cancer cells, minimizing harm to healthy tissues. In the case of cervical cancer, immunotherapy has proven to be particularly effective (9). Currently, the major categories of immunotherapy included oncolytic virus therapies, cancer vaccines, cytokine therapies, adoptive cell transfer, and immune checkpoint inhibitors, have evolved and shown promise in clinical practice (10).

Bibliometrics is a method used to analyze literature in a specific research field by evaluating the quantity and quality of publications (11, 12). This analysis provides a comprehensive understanding of the authors, keywords, journals, countries, institutions, and references related to the research field (12). Bibliometric analysis tools, such as CiteSpace (13), VoSviewer (12, 14), and bibliometrix in R package (15), allow for visualizing the results of the literature analysis. Recent research has demonstrated promising results for immunotherapy in the treatment of cervical cancer, suggesting it as a potential avenue for therapeutic advancement (16). Therefore, this study aims to conduct a bibliometric analysis of publications focused on immunotherapy in cervical cancer over the past twenty-four years, from 2000 to 2023. The objective is to identify the major contributors and current research status in this field, while also exploring future research trends and prospects for development.

## Methods

### Search strategy

**Figure 1:**
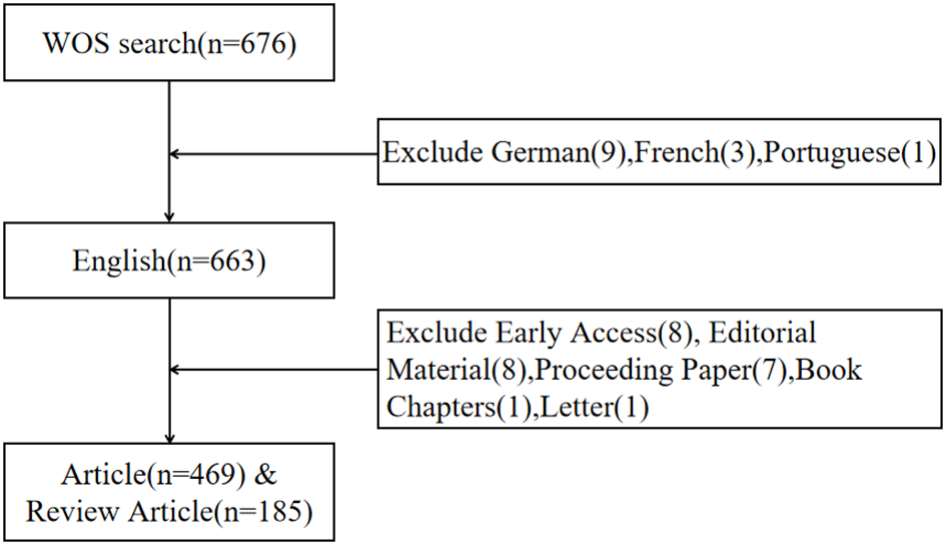
The study flow diagram for screening.

On August 01, 2023, we utilized the Web of Science Core Collection (WoSCC) database (https://www.webofscience.com/wos/woscc/basic-search) to conduct a comprehensive literature review on the topic of immunotherapy for cervical carcinoma. The time frame for our search was from the year 2000 to 2023. We employed a specific search formula to ensure the relevancy of our findings, which was ((TS = (cervical carcinoma)) AND TS = (immunotherapy)) AND LA = (English). Additionally, we restricted the search results to the document types of “articles” and “reviews”(**Figure 1**).

### Data analysis

VOSviewer (version 1.6.19) is a powerful bibliometric analysis software that allows researchers to extract key information from a vast number of publications (14, 17). This software is widely used for building collaboration, co-citation, and co-occurrence networks in various scientific fields (18). In our study, the software mainly completes the following analysis: country and institution analysis, journal and co-cited journal analysis, author and co-cited author analysis, and keyword co-occurrence analysis. In the map produced by VOSviewer, a node represents an item such as country, institution, journal, and author. Node size and color indicate the number and classification of these items, respectively. Line thickness between nodes reflects the degree of collaboration or co-citation of the items (19, 20).

CiteSpace (version 6.2.R4) is a software tool developed by Professors Chen C specifically designed for conducting bibliometric analysis and visualizing research data (13, 21). In our research study, we utilized CiteSpace to create a dual-map overlay, which allowed us to visualize the interconnections between different journals in our field of study. Additionally, we employed CiteSpace to analyze reference patterns using a feature called Citation Bursts. This feature enabled us to identify significant shifts or bursts in citation activity for specific references over time.

The R package “bibliometrix” (version 4.3.1) (https://www.bibliometrix.org) is an open-source software tool that provides a comprehensive set of functions for bibliometric analysis (22). It is designed to process and analyze large volumes of scientific literature data, primarily from academic journals. Additionally, Microsoft Office Excel was used to conduct quantitative analysis of publication.

## Results

### Quantitative analysis of publication

**Figure 2:**
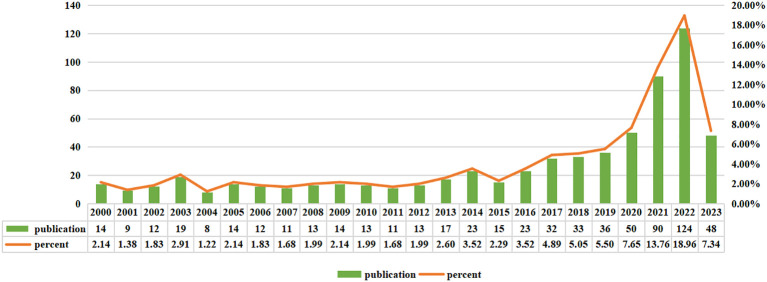
Annual output of research of immunotherapy in cervical carcinoma.

Based on the search strategy we employed, there were a total of 654 studies of immunotherapy in relation to cervical carcinoma over the course of the last 24 years, including 469 “articles” and 185 “reviews”. As shown in **Figure 2**, Judging from the growth rate of the number of publications each year, it can be observed that the field of immunotherapy in cervical carcinoma was in its early stages of research from 2000 to 2015. The annual publication volume was less than 20 articles and an average annual publication number of about 14. However, there has been a significant shift in the number of publications in the field from 2016 to 2022. The average annual publication number during this period was approximately 55, showcasing a substantial increase compared to previous years. The number of relevant publications published in 2022 was 124, 5.4 times that of 2016. Currently, 48 articles have been published in August 01 2023, and it is believed that a new peak will be reached by the end of the year.

### Country/regions and institutional analysis

**Figure 3:**
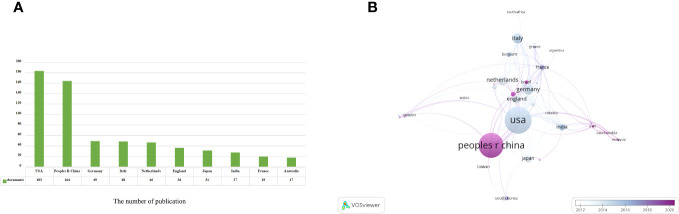
Analysis of countries/regions engaged in immunotherapy research in relation to cervical carcinoma. **(A)** The top 10 most productive countries/regions. **(B)** A network map showing countries/regions involved in the research on immunotherapy in relation to cervical carcinoma.

Based on the information provided, a total of 654 articles were collected from 66 different countries. According to the publishing number, as shown in the bar graph (**Figure 3A**), the top 3 countries/regions were United States (n = 183, 28.37%), China (n = 164, 25.08%) and Germany (n = 49, 7.60%), respectively. The combined number of publications from China and the United States accounted for almost half of the total (53.45%). Subsequently, we filtered and visualized 66 countries based on the number of publications more than or equal to 2, and constructed a collaborative network based on the number and relationship of publications in each country (**Figure 3B**). It is worth noting that this network illustrates a high level of active cooperation between different countries. For instance, China showed close collaboration with several countries, including the United States, Germany, Australia, Sweden, and France. Similarly, the United States was found to have active partnerships with Italy, Japan, India, England, and Iran.

**Figure 4:**
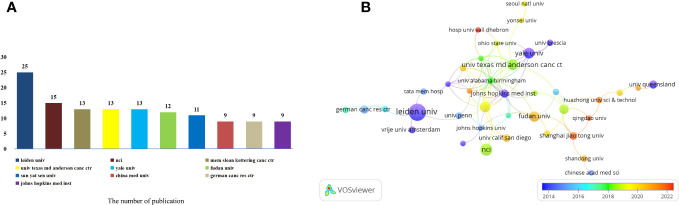
Analysis of institutions involved in immunotherapy research in relation to cervical carcinoma. **(A)** The top 10 institutions involved in immunotherapy research in relation to cervical carcinoma. **(B)** A network map showing institutions involved in immunotherapy research in relation to cervical carcinoma.

These articles were contributed by a total of 1080 institutions. Among them, the top 10 institutions played a significant role by contributing 129 articles, which accounted for 19.72% of all the articles (**Figure 4A**). Memorial Sloan-Kettering Cancer Center, The University of Texas MD Anderson Cancer Center and Yale University have an equal number of 13 published articles. China Medical University, German Cancer Research Center and Johns Hopkins Medical Institution also have the same number of published articles, both of which are 9. To further analyze the collaboration patterns, we selected 51 institutions based on the minimum number of publications equal to 5 for visualization, and constructed a collaborative network based on the number and relationship of publications of each institution. As shown in **Figure 4B**, we observed a close cooperation between Leiden University, Memorial Sloan-Kettering Cancer Center, German Cancer Research Center and Vrije Universiteit Amsterdam. Additionally, there was active collaboration between Fudan University, Shanghai Jiao Tong University, Johns Hopkins Medical Institution and National University Of Singapore. In another interesting finding, we note that the cooperation between Sun Yat Sen University, The University of Texas MD Anderson Cancer Center, Huazhong University of Science And Technology and Qingdao University is very close. Further analysis of the research patterns in different years revealed that Leiden University, Johns Hopkins Medical Institution, Yale University, Johns Hopkins Medical Institution and The University Of Queensland conducted extensive research in 2014. On the other hand, Fudan University, Shanghai Jiao Tong University and Qingdao University have been extensively studied in 2022.

### Journals and co-cited journals

**Figure 5:**
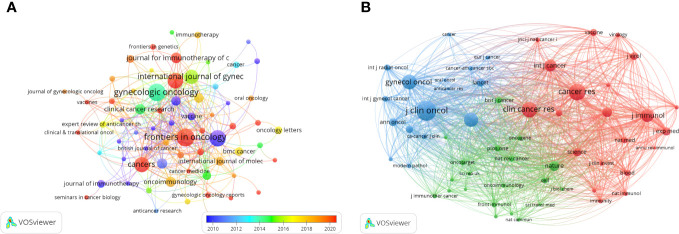
The visualization of journals **(A)** and co-cited journals **(B)** on research of immunotherapy in cervical carcinoma.

Publications related to immunotherapy in cervical carcinoma were published in a total of 293 different journals. Among these journals, Frontiers in Oncology had the highest number of published papers (n=21, 7.17%), followed by Gynecologic Oncology (n=20, 6.83%), International Journal Of Cancer (n=18, 6.14%), Frontiers in Immunology (n=17, 5.8%) and Cancers (n=16, 5.46%). In addition, among the top 10 journals, the journal with the highest impact factor is Clinical Cancer Research (IF=11.5), followed by journal for Immunotherapy of Cancer (IF=10.9). Following this analysis, we screened 72 journals based on the minimum number of relevant publications equal to 3 and mapped the journal network (**Figure 5A**). This visualization reveals that Frontiers in Oncology has active citation relationships with several other journals, including Gynecologic Oncology, Cancers, and Clinical Cancer Research, etc.

Based on the information provided in the quote, **Table 1** shows the top 10 co-cited journals, five journals were cited more than 1000 times, and Journal OF Clinical Oncology (Co-citation=1718) was the most cited journal, followed by Cancer Research (Co-citation=1346), Clinical Cancer Research (Co-citation=1205), Gynecologic Oncology (Co-citation=1234) and New England Journal Of Medicine (Co-citation=1077). In addition, the impact factor of New England Journal OF Medicine is the highest (IF=158.5), followed by Nature (IF=64.8). To explore the co-citation network further, journals with a minimum co-citation equal to 120 were filtered and mapped in **Figure 5B**. From this figure, it can be observed that Journal OF Clinical Oncology has positive co-citation relationships with journals such as New England Journal OF Medicine, Gynecologic Oncology and International Journal Of Gynecological Cancer, etc.

**Table 1:** Top 10 journals and co-cited journals for research of immunotherapy in cervical carcinoma.

Rank	Journal	Count	IF	Q	Co-cite Journal	Co-citation	IF	Q
1	Frontiers In Oncology	21 (7.17%)	4.7	Q2	Journal OF Clinical Oncology	1718	45.3	Q1
2	Gynecologic Oncology	20 (6.83%)	4.97	Q2	Cancer Research	1346	11.2	Q1
3	International Journal Of Cancer	18 (6.14%)	6.4	Q1	Clinical Cancer Research	1205	11.5	Q1
4	Frontiers In Immunology	17 (5.80%)	7.3	Q1	Gynecologic Oncology	1134	4.97	Q2
5	Cancers	16 (5.46%)	5.2	Q1	New England Journal Of Medicine	1077	158.5	Q1
6	International Journal Of Gynecological Cancer	15 (5.12%)	4.8	Q1	Journal Of Immunology	975	4.4	Q2
7	Journal For Immunotherapy Of Cancer	10 (3.41%)	10.9	Q1	International Journal Of Cancer	825	6.4	Q1
8	Cancer Immunology Immunotherapy	9 (3.07%)	5.8	Q1	Nature	642	64.8	Q1
9	Clinical Cancer Research	9 (3.07%)	11.5	Q1	Proceedings Of The National Academy Of Sciences Of The United States Of America	632	11.1	Q1
10	Oncoimmunology	9 (3.07%)	7.69	Q1	Journal Of Experimental Medicine	516	15.3	Q1

**Figure 6:**
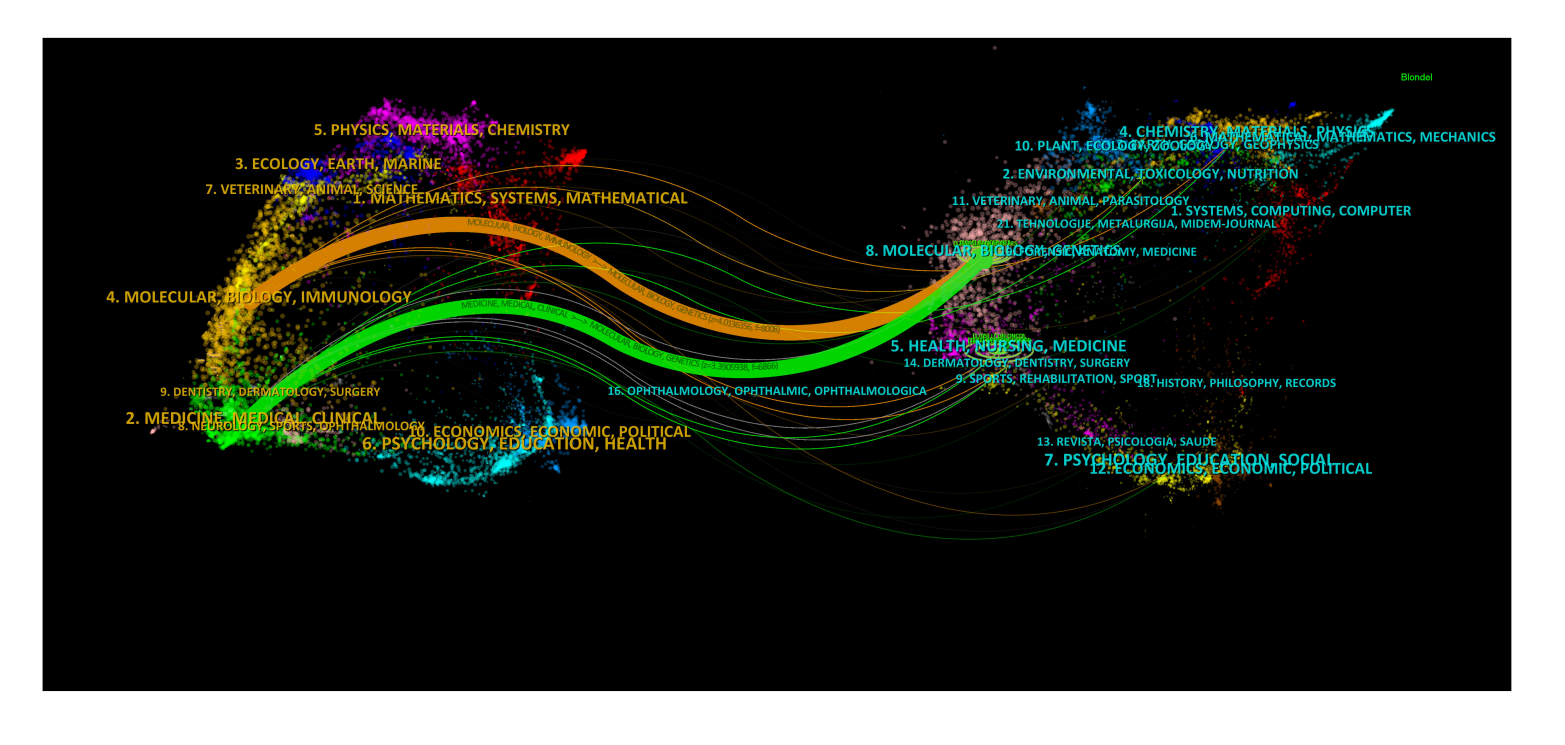
The dual-map overlay of journals on research of immunotherapy in cervical carcinoma.

The dual-map overlay of journals in **Figure 6** demonstrated the topic distribution of the journals. The location of the citing journals was on the left side of the map, while the cited journals were located on the right side. The labels on the map represented the different disciplines covered by the journals. From left to right, the colored lines depicted the citation paths. There were two distinct citation paths. The orange citation path suggested that studies from Molecular/Biology/Genetics journals were frequently cited in studies from Molecular/Biological/Immunology journals. On the other hand, the green path suggested that studies from Molecular/Biological/Genetic journals were frequently cited in studies from Medicine/Medical/Clinical journals.

### Authors and co-cited authors

**Figure 7:**
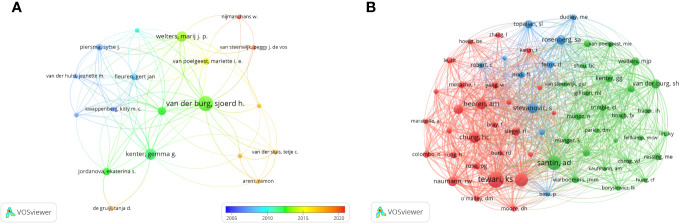
The visualization of authors **(A)** and co-cited Authors **(B)** on research of immunotherapy in cervical carcinoma.

In the research of immunotherapy in cervical carcinoma, a total of 3952 authors participated. The top three authors with the most publications were Van Der Burg, Sjoerd h (n=15), Kenter, Gemma g (n=10) and Welters, Marij j.p (n=9) (**Table 2**). We build a collaborative network based on authors whose number of published papers is more than or equal to 3 (**Figure 7A**). The three authors mentioned earlier have the largest nodes because they publish the most related publications. Furthermore, the collaborative network revealed that there were close collaborations among multiple authors. For example, Melief, Cornelis j. m has close cooperation with Kenter, Gemma g, Jordanova, Ekaterina s and Welters, Marij j. p, etc.

**Table 2:** Top 10 authors and co-cited authors on research of immunotherapy in cervical carcinoma.

Rank	Authors	Count	Co-Cited Authors	Citations
1	Van Der Burg, Sjoerd h.	15	Tewari, Krishnansu s.	167
2	Kenter, Gemma g.	10	Santin, Alessandro d.	137
3	Welters, Marij j. p.	9	Monk, Bradley j.	131
4	Santin, Alessandro d.	8	Chung, Hung-Chang	101
5	Wu, Tc	8	Heeren, Anne Marijne	96
6	Melief, Cornelis j. m.	7	Stevanovic, Sinisa	92
7	Hinrichs, Christian s.	6	Frenel, Jean-Sebastien	83
8	Jordanova, Ekaterina s.	6	Naumann, R. Wendel	80
9	Monk, Bradley j.	6	Rosenberg, Steven A.	80
10	Tewari, Krishnansu s.	6	Van Der Burg, Sjoerd h.	78

In terms of co-citations, among the 20271 co-cited authors, four authors were co-cited more than 100 times (**Table 2**). The most co-cited author is Tewari, Krishnansu Sujata (n=167), followed by Santin, Alessandro (n=137), Monk, Bradley J (n=131) and Chung, Hung-Chang (n=101). Authors with minimum co-citations equal to 30 were filtered to map co-citation network graphs. As shown in **Figure 7B**, there are also active collaborations among different co-cited authors, such as Tewari, Krishnansu Sujata and Chung, Hung-Chang, Naumann, R. Wendel and Monk, Bradley J.

### Co-cited references

**Figure 8:**
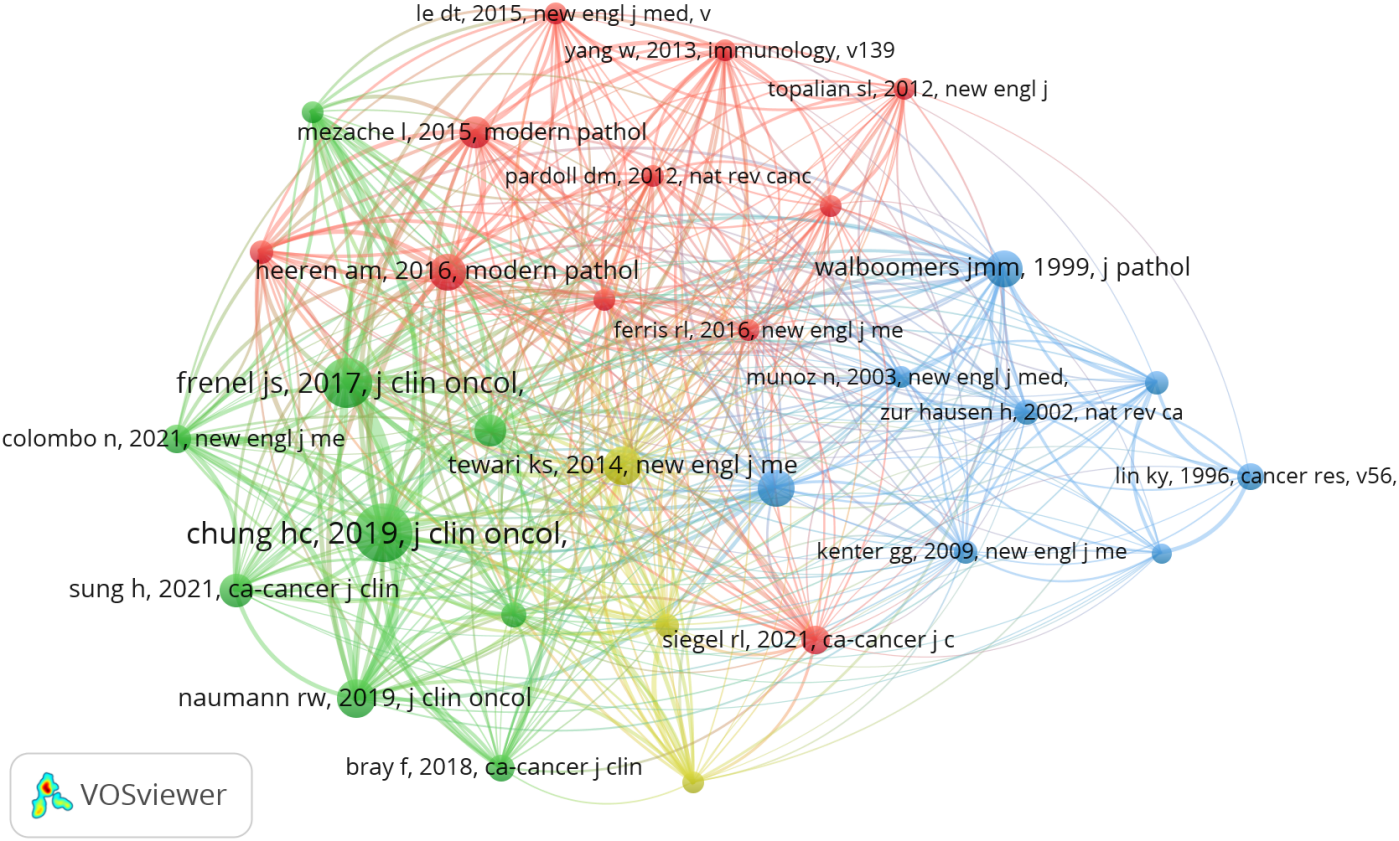
The visualization of co-cited references on research of immunotherapy in cervical carcinoma.

There are 27909 co-cited references on research of immunotherapy in cervical carcinoma over the past 24 years. Among the top 10 co-cited references in this area (**Table 3**), all references were co-cited at least 48 times, and two references was co-cited more than 70 times. For the construction of the co-citation network map, we specifically selected references that were co-cited 30 times or more. According to **Figure 8**, one notable relationship that stands out “chung hc, 2019, j clin oncol” shows active co-cited relationships with “frenel js, 2017, j clin oncol”, “tewari ks, 2017, lancet” and “naumann rw, 2019, j clin oncol”, etc.

**Table 3:** Top 10 co-cited references on research of immunotherapy in cervical carcinoma.

Rank	co-cited reference	citations
1	chung hc, 2019, j clin oncol, v37, p1470	87
2	frenel js, 2017, j clin oncol, v35, p4035	74
3	tewari ks, 2014, new engl j med, v370, p734	57
4	naumann rw, 2019, j clin oncol, v37, p2825	56
5	heeren am, 2016, modern pathol, v29, p753	55
6	stevanovic s, 2015, j clin oncol, v33, p1543	55
7	walboomers jmm, 1999, j pathol, v189, p12	53
8	sung h, 2021, ca-cancer j clin, v71, p209	50
9	mezache l, 2015, modern pathol, v28, p1594	48
10	tewari ks, 2017, lancet, v390, p1654	48

### Reference with citation bursts

**Figure 9:**
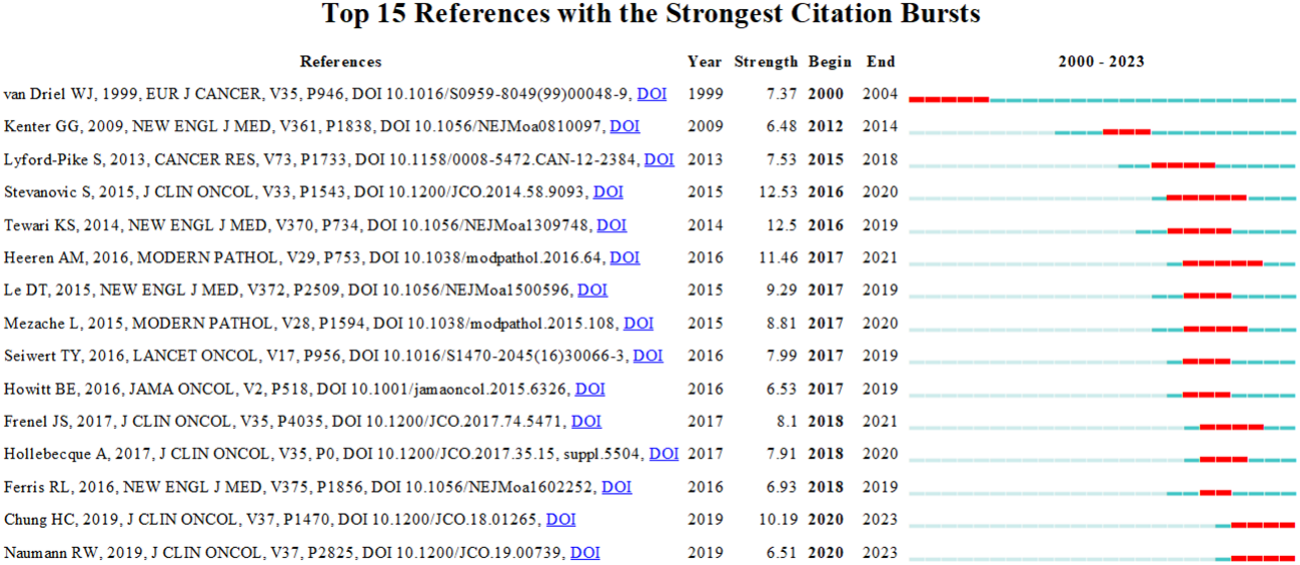
Top 15 references with strong citation bursts. A red bar indicates high citations in that year.

Reference with citation bursts refers to those references that are frequently cited by scholars in a certain field over a period of time. Based on our analysis using CiteSpace, we were able to identify 15 references that exhibited strong citation bursts in our study. As shown in **Figure 9**, every bar represents a year, and the red bar represents strong citation burstiness. The analysis revealed that citation bursts for these references started as early as 2000 and continued until 2020. The reference with the strongest citation burst (strength=12.53) was titled “Complete Regression of Metastatic Cervical Cancer After Treatment With Human Papillomavirus–Targeted Tumor-Infiltrating T Cells”, published in Journal Of Clinical Onology by Sanja Stevanović et al. with citation bursts from 2016 to 2020. The second strongest citation burst (strength=12.5) was observed in a reference titled “Improved Survival with Bevacizumab in Advanced Cervical Cancer”, published in New England Journal OF Medicine by Krishnansu S. Tewari et al. In general, the bursts strength of these 15 references ranged from 6.48 to 12.53. Additionally, the endurance strength of these bursts ranged from 2 to 5 years. **Table 4** summarizes the main research contents of the 15 references in the order of the literature in **Figure 9**.

**Table 4:** The main research contents of the 15 references with strong citations bursts.

Rank	strength	Main research content
1	7.37	Vaccination with HPV16 peptides of patients with advanced cervical carcinoma: clinical evaluation of a phase I–II trial
2	6.48	Vaccination against HPV-16 Oncoproteins for Vulvar Intraepithelial Neoplasia
3	7.53	Evidence for a Role of the PD-1:PD-L1 Pathway in Immune Resistance of HPV-Associated Head and Neck Squamous Cell Carcinoma
4	12.53	Complete Regression of Metastatic Cervical Cancer After Treatment With Human Papillomavirus–Targeted Tumor-Infiltrating T Cells
5	12.5	Improved Survival with Bevacizumab in Advanced Cervical Cancer
6	11.46	Prognostic effect of different PD-L1 expression patterns in squamous cell carcinoma and adenocarcinoma of the cervix
7	9.29	PD-1 Blockade in Tumors with Mismatch-Repair Deficiency
8	8.81	Enhanced expression of PD L1 in cervical intraepithelial neoplasia and cervical cancers
9	7.99	Safety and clinical activity of pembrolizumab for treatment of recurrent or metastatic squamous cell carcinoma of the head and neck (KEYNOTE-012): an open-label, multicentre, phase 1b trial
10	6.53	Genetic Basis for PD-L1 Expression in Squamous Cell Carcinomas of the Cervix and Vulva
11	8.1	Safety and Efficacy of Pembrolizumab in Advanced, Programmed Death Ligand 1–Positive Cervical Cancer: Results From the Phase Ib KEYNOTE-028 Trial
12	7.91	An open-label, multicohort, phase I/II study of nivolumab in patients with virus-associated tumors (CheckMate 358): Efficacy and safety in recurrent or metastatic (R/M) cervical, vaginal, and vulvar cancers
13	6.93	Nivolumab for Recurrent Squamous-Cell Carcinoma of the Head and Neck
14	10.19	Efficacy and Safety of Pembrolizumab in Previously Treated Advanced Cervical Cancer: Results From the Phase II KEYNOTE-158 Study
15	6.51	Safety and Efficacy of Nivolumab Monotherapy in Recurrent or Metastatic Cervical, Vaginal, or Vulvar Carcinoma: Results From the Phase I/II CheckMate 358 Trial

### Hotspots and frontiers

**Figure 10:**
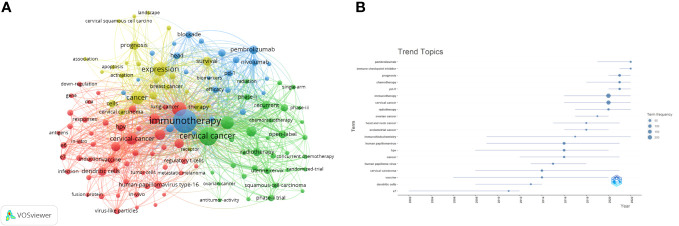
Keyword cluster analysis **(A)** and trend topic analysis **(B)**.

Through the co-occurrence analysis of keywords, we could quickly capture research hotspots in a certain field. **Table 5** presents the top 20 high-frequency keywords in research of immunotherapy in cervical carcinoma. It was observed that keywords like squamous-cell carcinoma, human-papillomavirus, and t-cells appeared more than 60 times, indicating that these areas were at the forefront of immunotherapy research for cervical carcinoma. We filtered keywords with the number of occurrences more than or equal to 10 and performed cluster analysis through VOSviewer (**Figure 10A**). The strength of the connections between keywords was represented by the thickness of the lines. The thicker the lines between the nodes, the stronger the connection between the keywords. As shown in **Figure 10A**, we obtained four clusters in total, representing four research directions. The keywords in the green cluster included cervical cancer, radiotherapy, chemotherapy, squamous-cell carcinoma, etc. The red cluster contained keywords such as human papillomavirus, vaccine, dendritic cells, cancer immunotherapy, etc. The keywords in blue clusters consist of biomarkers, pembrolizumab, pd-l1, nivolumab, etc. Lastly, the keywords in yellow clusters consist of expression, prognosis, survival, apoptosis, etc.

**Table 5:** Top 20 keywords on research of immunotherapy in cervical carcinoma.

Rank	Keyword	Count	Rank	Keyword	Count
1	immunotherapy	337	11	human papillomavirus	53
2	cervical cancer	200	12	survival	53
3	carcinoma	125	13	tumor-infiltrating lymphocytes	50
4	expression	115	14	dendritic cells	47
5	cancer	101	15	prognosis	47
6	squamous-cell carcinoma	100	16	hpv	46
7	cervical-cancer	81	17	pembrolizumab	45
8	human-papillomavirus	66	18	therapy	44
9	t-cells	65	19	recurrent	43
10	chemotherapy	59	20	radiotherapy	40

The trend topic analysis of the keywords (**Figure 10B**) showed that from 2002 to 2011, the research in this period mainly focused on searching for the pathogenic factors causing Cervical Cancer. Since 2012, there was a significant shift towards the development of vaccines for cervical carcinoma, as reflected by the prominence of keywords like HPV, vaccine, human papilloma virus, etc. In recent years, the primary focus of cervical carcinoma research has shifted towards immunotherapy, with keywords like immunotherapy, pd-l1, chemotherapy and immune checkpoint inhibitor gaining significant attention.

## Discussion

### General information

The present study conducted a comprehensive literature search on the Web of Science databases to identify articles published over the past 24 years (2000-2023) regarding immunotherapy for cervical carcinoma. To ensure the inclusion of only relevant and reliable studies, we applied stringent screening criteria and eliminated articles that did not meet these predetermined standards. Consequently, this scientometric study comprised 654 English papers published in 293 journals with 27909 co-cited references from 1080 institutions in 66 countries/regions. This signifies the interconnectedness and collaboration within the scientific community regarding immunotherapy for cervical carcinoma.

From the analysis of the results, it is evident that there has been a consistent increase in the number of publications related to immunotherapy for cervical carcinoma. This upward trend indicates that the scientific community has paid considerable attention to this particular topic in recent years. Looking at the growth rate of publications over the years, it becomes apparent that the field of immunotherapy for cervical carcinoma was at its early stages of research between 2000 and 2015. During this period, there was limited interest and attention given to exploring immunotherapy as a potential treatment option for cervical carcinoma. However, there has been a remarkable shift in the number of publications in this field from 2016 to 2023. The average annual publication number witnessed a significant increase compared to the previous years, suggesting a growing interest and recognition of the potential of immunotherapy in treating cervical carcinoma.

The United States and China are leading the way in conducting research on immunotherapy in cervical carcinoma. These two countries together contribute almost half of the total number of publications in this field. The United States is particularly dominant, with 50% of the top 10 research institutions being located there. China follows closely behind with three institutions, accounting for 30% of the top 10. The Netherlands and Germany each have one institution, making up 10% of the top 10 each. A noteworthy observation is the close cooperation among six countries: the United States, China, Germany, Australia, Sweden, and France. This indicates that there is a collaborative effort among these nations to drive progress in the field of immunotherapy for cervical carcinoma.

Furthermore, specific research institutions have established strong collaborative relationships, Leiden University, Memorial Sloan-Kettering Cancer Center, German Cancer Research Center and Vrije Universiteit Amsterdam. These institutions actively work together to advance research in this area. Additionally, there was active collaboration between Fudan University, Shanghai Jiao Tong University, Johns Hopkins Medical Institution and National University Of Singapore. In another finding, we note that the cooperation between Sun Yat Sen University, The University of Texas MD Anderson Cancer Center, Huazhong University of Science And Technology and Qingdao University is very close. This highlights the strong cooperation between these institutions. Analyzing research patterns over the years, it is evident that the United States and the Netherlands started conducting research relatively early in this field. However, China has shown a significant increase in research momentum in recent years. Despite cooperative relations among some countries, the breadth and intensity of cooperation between institutions are not ideal. For example, China has no cooperation with Leiden University from Netherlands with the highest number of publications. This lack of collaboration could potentially hinder the long-term development of research in this field. Therefore, it is strongly recommended that research institutions in different countries engage in extensive cooperation and communication to jointly promote the development of immunotherapy in cervical carcinoma. By working together, the advancements in this field can be accelerated, leading to improved treatment options and ultimately better outcomes for patients.

The publication of extensive research on immunotherapy in cervical carcinoma in Frontiers in Oncology (IF=4.7, Q2) suggests that this journal is highly sought after in the field of cervical cancer research. Moreover, among the various journals, Clinical Cancer Research (IF=11.5, Q1) boasts the highest impact factor, indicating its great significance in the field. Following closely is the Journal for Immunotherapy of Cancer (IF=10.9, Q1). Regarding the co-cited journals, we could find most of them are high-impact Q1 journals. Obviously, these journals are high-quality international journals, providing support for the study of immunotherapy in cervical carcinoma. Moreover, the current research on immunotherapy in cervical carcinoma is mostly published in journals related to Molecular/Biological/Immunology and Medicine/Medical/Clinical disciplines. This suggests that we are presently in a phase of mutual transformation, bridging the gap between fundamental research and clinical implementation. It is an encouraging sign that advancements are being made to conquer cervical cancer, and we hold firm belief that one day we will achieve complete success in our battle against this disease.

According to the author’s perspective, it has been observed that each of the top ten active authors has published a minimum of 6 articles. Two authors in particular, Van Der Burg, Sjoerd h and Kenter, Gemma g have published more than 10 articles, indicating their dedication and prolific contributions to the field of cervical carcinoma immunotherapy. Interestingly, it was found that more than half of the authors involved in this research were from the Netherlands. This suggests that Netherlands researchers are prominently involved in cervical carcinoma immunotherapy research and have made substantial contributions to the field. Their expertise and dedication may have played a significant role in advancing potential treatments and approaches for cervical cancer. Furthermore, when analyzing co-citations, it was revealed that the top 10 authors with a minimum of 78 co-citations have made significant contributions to the field of cervical carcinoma immunotherapy. Tewari, Krishnansu S (167 co-citations) ranked first, followed by Santin, Alessandro D (137 co-citations) and Monk, Bradley J (131 co-citations). Tewari, Krishnansu S have achieved significant results in targeted therapy (23), chemotherapy (24, 25), clinical prognosis (26) and survival research (27) for cervical cancer. His contributions have propelled the field forward and provided valuable insights into potential treatment approaches. Santin, Alessandro D from Yale University conducted research on targeted therapy for cervical cancer and led a clinical study on the therapeutic effects of combination of multiple drugs for cervical cancer treatment (28, 29). His research has opened up new possibilities for more effective and personalized treatment options. Monk, Bradley J from the University of Arizona studied topotecan and paclitaxel, tisotumab vedotin (TV), pembrolizumab and lenvatinib for the treatment of cervical cancer (30, 31). In the era of precision medicine, his research provides new treatment options for the treatment of cervical cancer. Several articles show that Monk, Bradley J and Tewari, Krishnansu S cooperate to publish the treatment of cervical cancer. This active cooperative research is playing a crucial role in driving breakthroughs in the treatment of cervical cancer, showing the benefits of collaborations in advancing medical knowledge and improving patient outcomes.

### Knowledge base

A co-cited reference is a type of reference that appears in multiple publications, indicating that it serves as the foundation or basis for research in a particular field (32). In this bibliometric study, we selected the 10 co-cited references with the highest number of co-citations to determine the research basis of immunotherapy in cervical carcinoma. In 1999, Walboomers Jmm discovered that human papillomavirus (HPV) is a necessary cause of invasive cervical cancer worldwide (33, 34). This groundbreaking discovery led to significant advancements in the understanding and prevention of cervical cancer, as well as the development of vaccines to prevent its spread. Since then, numerous studies have confirmed the role of HPV in the development of cervical cancer and efforts have been made to increase global vaccination rates to reduce the incidence of this deadly disease. On August 14, 2014, the United States Food and Drug Administration approved the anti-angiogenesis drug, bevacizumab, for women with advanced cervical cancer based on a 2012 interim analysis of 271 deaths on GOG protocol 240 (35). Krishnansu S Tewari evaluated the effectiveness of bevacizumab and nonplatinum combination chemotherapy in patients with recurrent, persistent, or metastatic cervical cancer in 2014 (35). The aim of the study was to determine whether the addition of bevacizumab to the chemotherapy regimen would provide any significant benefits to these patients. Fast forward to 2017, The study showed that the benefit conferred by incorporation of bevacizumab is sustained with extended follow-up as evidenced by the overall survival curves remaining separated (36). In 2015, Sanja Stevanović made a groundbreaking discovery by developing a method to generate T-cell cultures from HPV-positive cancers. These T-cell cultures were specifically selected to react to the HPV oncoprotein (37). The aim was to administer these cultures to patients and study if they could induce cancer regression. The final discovery that durable, complete regression of metastatic cervical cancer can occur after a single infusion of HPV-TILs (37, 38). This breakthrough opened up new possibilities in the field of immunotherapy for cervical cancer, offering hope to patients who were previously left with limited treatment options.

Another aspect that played a crucial role in the development of effective immunotherapies for cervical cancer is the discovery of programmed death ligand 1 (PD L1) (39). PD L1 is a protein with the ability to greatly influence the adaptive arm of the immune system (37). It can bind to its ligands, including PD 1 and CD80, thereby inhibiting the proliferation and activity of cytotoxic CD8 T cells that respond to viral or cancer-associated antigens (40). Increased PD L1 expression may allow viruses to avoid immune surveillance (41, 42). In 2015, Louisa Mezache conducted a study that shed light on the significance of PD L1 in cervical cancer. The research demonstrated that PD L1 is a reliable biomarker for productive HPV infection in the cervix (40). Furthermore, PD L1 expression was found to be significantly upregulated in both the carcinoma and surrounding inflammatory cells in cervical cancer compared to other gynecologic malignancies (43). This finding suggests that anti-PD L1 therapy may have great potential in the treatment of cervical cancer. In 2016, A Marijne Heeren findings point to a key role of PD-L1 in immune escape of cervical cancer and provide a rationale for therapeutic targeting of the PD-1/PD-L1 pathway (41). One specific immunotherapy called pembrolizumab has emerged as a highly promising treatment option. Pembrolizumab is a highly selective, fully humanized monoclonal antibody that prevents the interaction between PD-1 and its ligands, programmed death ligand 1 (PD-L1) and programmed death ligand 2 (PD-L2) (9, 44). By inhibiting this interaction, pembrolizumab helps to restore the immune system’s ability to recognize and attack cancer cells in cervical cancer patients. Owing to its success and proven efficacy, pembrolizumab has received approval for use in treating melanoma, a form of skin cancer (45). In 2017, Jean-Sebastien Frenel managed the KEYNOTE-028 trial, which was specifically designed to evaluate the safety and effectiveness of pembrolizumab in 20 cohorts of advanced solid tumors that tested positive for programmed death ligand 1 (46). The final conclusion shows that in patients with programmed death ligand 1–positive advanced cervical cancer, pembrolizumab demonstrated antitumor activity and exhibited a safety profile consistent with that seen in other tumor types (46). In June 2018, pembrolizumab also received accelerated approval for the treatment of patients with recurrent/metastatic cervical cancers expressing programmed death-ligand 1 (PD-L1) after receiving chemotherapy. In 2019, Hyun Cheol Chung led the KEYNOTE-158 study, a phase II investigation aiming to assess the antitumor activity and safety of pembrolizumab across multiple cancer types. In the study, pembrolizumab monotherapy exhibited durable antitumor activity and managed safety in patients with advanced cervical cancer (47). On the basis of these results, the US Food and Drug Administration granted accelerated approval of pembrolizumab for patients with advanced PD-L1–positive cervical cancer who experienced progression during or after chemotherapy (47). Nivolumab is a fully human immunoglobulin G4 programmed death-1 immune checkpoint inhibitor that is approved for the treatment of various cancers (48, 49). In the ongoing phase I/II study called CheckMate 358, which evaluates nivolumab-based therapy in virus-associated tumors. R Wendel Naumann reported that the efficacy of nivolumab in patients with recurrent/metastatic cervical, vaginal, or vulvar cancers is highly promising and justifies further investigation (50).

In conclusion, the highly co-cited references reviewed here provide an extensive and insightful examination of the current state of cervical cancer epidemiology, the advancements in immunotherapy for advanced cervical cancer and the clinical implementation of these treatments. Co-citation analysis can provide us with a wealth of useful information, allowing us to gain a better understanding of the evolution of the knowledge structure relating to cervical cancer immunotherapy.

### Hotspots and frontiers

References with citation bursts indicate emerging topics within this research field, as researchers have frequently cited these references in their publications (51). The main research contents of these highly-cited references shed light on the critical areas of study in the context of immunotherapy for cervical carcinoma. Firstly, studying the biological role of immunotherapy in cervical carcinoma is a major focus. This includes investigating the interactions between cancer cells and the immune system, identifying key molecules involved in immune responses, and exploring strategies to enhance the body’s natural defense mechanisms against cervical carcinoma. Secondly, the pathogenesis of immunotherapy in cervical carcinoma is another crucial area of research. Understanding the underlying pathways and molecular processes involved in the response to immunotherapy can provide valuable insights into developing novel therapeutic strategies for managing cervical carcinoma. Lastly, researchers are actively exploring the potential of immune-related drugs in the treatment of cervical carcinoma. This involves investigating the efficacy and safety of immunotherapeutic agents specifically designed to target the unique characteristics of cervical cancer cells. By studying the use of immune-related drugs, researchers hope to develop more effective treatment options that can improve patient outcomes and reduce the side effects associated with traditional therapies.

In addition to references with citation bursts, keywords can also help us quickly capture the distribution and evolution of hotspots in the research field of immunotherapy in cervical carcinoma. Excluding keywords such as immunotherapy, cervical cancer and expression, **Table 5** mainly includes the following keywords: t-cells, tumor-infiltrating lymphocytes, dendritic cells, pembrolizumab and recurrent. According to keyword clustering analysis and trend topic analysis (**Figure 10**), we concluded that the research of immunotherapy in cervical carcinoma mainly focuses on the following aspects:

### PD-1/PD-L1

The PD-1/PD-L1 pathway plays a crucial role in regulating the immune response within the tumor microenvironment. It controls the process of immune tolerance, which allows cancer cells to evade detection and destruction by the immune system (52). The activity of PD-1 and its ligands PD-L1 or PD-L2 are responsible for T cell activation, proliferation, and cytotoxic secretion in cancer to degenerating anti-tumor immune responses (53).

PD-1, also referred to as CD279, is a 55-kDa transmembrane protein containing 288 amino acids with an extracellular N-terminal domain (IgV-Like), a membrane-permeating domain and a cytoplasmic tail located at the N and C ends, respectively, with two tyrosine base (54). It is an inhibitor of both adaptive and innate immune responses, and is expressed on activated T, natural killer (NK) and B lymphocytes, macrophages, dendritic cells (DCs) and monocytes (55). Of note, It is highly expressed on tumor-specific T cells. PD-1 plays a dual role in the immune system, functioning both as a beneficial regulator and a harmful promoter. On one hand, it plays a crucial role in reducing the regulation of ineffective or harmful immune responses, ensuring the body’s immune system functions properly. This helps maintain immune tolerance and prevents autoimmune diseases. However, on the other hand, PD-1 can also have detrimental effects. It causes the dilation of malignant cells by interfering with the protective immune response (56).

PD-1 ligand (PD-L1, also referred to as CD279 and B7-H1), a protein that binds to PD-1, belongs to the B7 series and is a 33-kDa type 1 transmembrane glycoprotein that contains 290 amino acids with Ig- and IgC domains in its extracellular region (57). It is expressed by tumor cells as an “adaptive immune mechanism” to escape anti-tumor responses. It acts as a pro-tumorigenic factor in cancer cells via binding to its receptors and activating proliferative and survival signaling pathways (58, 59). In addition, PD-L1 has been shown to exert non-immune proliferative effects on a variety of tumor cell types. For example, it can induce epithelial-to-mesenchymal transition (EMT) and stem cell-like phenotypes in renal cancer cells. This suggests that the intrinsic pathway of PD-L1 can further enhance the progression of kidney cancer (60).

In summary, while PD-1 has beneficial effects in regulating the immune system, it can also cause harm by aiding the growth and survival of cancer cells. PD-L1, as a key player in this process, helps tumor cells evade the body’s anti-tumor responses and promotes cancer progression.

### Immune checkpoint inhibitor

Immune checkpoint inhibitors (ICIs) have emerged as highly effective therapies for many cancers. These therapies work by blocking specific proteins on immune cells that normally act as brakes to prevent an immune response from becoming too strong and causing damage to healthy tissues (61, 62). Thus far, all approved ICIs are monoclonal antibodies that block cytotoxic T lymphocyte–associated protein 4 (CTLA-4), programmed cell death protein-1 (PD-1) or programmed death-ligand 1 (PD-L1), each a key inhibitor of T cell activation and function (63, 64). As of December 2020, seven immunotherapy drugs have received approval from the Food and Drug Administration (FDA). The significant increase in the number of ICI trials from 2250 in 2018 to 3428 in 2019 demonstrates the growing importance of ICIs in cancer treatment (65, 66). Overall, the development of ICIs and the understanding of the mechanisms behind their efficacy have paved the way for new strategies to enhance the immune response against cancer. The relationship between tumor cell expression of immune checkpoint proteins and response to treatment is complex and ongoing research aims to further unravel its intricacies.

### Anti-TGF-β/PD-L1 bispecific antibody

Despite anti-PD-1/PD-L1 monoclonal antibodies being approved for treating multiple malignancies and showing promising anti-tumor effects in some patients, there is still a challenge due to the low objective response rate in patients. Several studies have found that the activity of the TGF-β pathway is significantly increased in immunotherapy-resistant tumors (67, 68). Transforming growth factor-beta (TGF-β) is a versatile cytokine that regulates various components in the cancer-immunity cycle. It inhibits T cell proliferation and activation, impairs the activities of dendritic cells (DC) and natural killer (NK) cells, promotes the differentiation of regulatory T (Treg) cells, and enhances the activities of cancer-associated fibroblasts (CAF) (69–71). The immunosuppressive mechanisms of TGF-β and PD-1 pathways are independent and complementary to each other, working together to help tumors evade the host immune surveillance. In order to address this challenge, a novel bifunctional fusion protein called M7824 has been developed. M7824 simultaneously targets TGF-β and PD-L1, blocking the immune escape mechanism of tumor cells and reducing the inhibition of the tumor microenvironment on immune cells (72–74). This promotes the immune system’s attack on the tumor and achieves an anti-tumor effect. Subsequently, more bispecific antibodies (BsAbs) like YM101 and BiTP have been developed. These antibodies also demonstrate potent anti-tumor activities in both preclinical studies.

YM101 is a bispecific antibody that blocks both TGF-β and murine PD-L1 based on the Check-BODY™ technology platform (75). *In vivo* experiments showed that YM101 has a stronger anti-tumor effect compared to using anti-TGF-β or anti-PD-L1 as single therapies. This is because YM101 promotes the formation of inflamed tumors by increasing the number and activity of tumor-infiltrating lymphocytes (TIL) and dendritic cells (DC). It also increases the ratio of M1/M2 macrophages, which are associated with tumor suppression. Moreover, YM101 has the ability to suppress the functions of cancer-associated fibroblasts (CAFs) and weaken the peritumoral barrier by neutralizing TGF-β in the tumor microenvironment (TME). This means that YM101 can improve the infiltration of T cells into the tumor and overcome resistance to anti-PD-L1 therapy (75, 76).

Based on the success of YM101 in the pilot study, a new bispecific antibody called BiTP was developed using the Check-BODY platform (77). BiTP targets both TGF-β and human PD-L1. *In vitro* experiments confirmed that BiTP effectively blocks the TGF-β-Smad and PD-L1-PD-1-NFAT signaling pathways. *In vivo* animal experiments further demonstrated that BiTP has superior antitumor activity compared to using anti-PD-L1 or anti-TGF-β alone. This is because BiTP reduces collagen deposition, enhances the penetration of CD8+ T cells into the tumor, and increases the presence of tumor-infiltrating lymphocytes. This overall improvement in the tumor microenvironment contributes to the strong antitumor effects of BiTP (77).

### Advantages and shortcomings

This study has several unique advantages. Firstly, we conducted a systematic analysis of research on immunotherapy in cervical carcinoma, which is the first of its kind using bibliometrics. This approach provides a comprehensive overview and guidance for scholars who are interested in studying this topic. By analyzing a large body of literature using bibliometric tools, we were able to identify the key trends, patterns and gaps in the research, which can help guide future studies and decision-making. Secondly, we utilized three different bibliometric tools simultaneously for the survey. Two of these tools, VOSviewer and CiteSpace, are widely recognized and extensively used in the field of bibliometrics (78). This choice of tools increases the validity and objectivity of our data analysis process. By using multiple tools, we were able to cross-validate the results and ensure the reliability of our findings. Finally, bibliometric analysis offers a more comprehensive insight into the hotspots and frontiers of research on immunotherapy in cervical carcinoma compared to traditional literature reviews. Traditional reviews often rely on manual selection and interpretation of a small number of papers, which may result in a biased and limited understanding. In contrast, bibliometric analysis allows for a broader and more systematic examination of the research landscape, providing a more complete picture of the current state of knowledge, emerging trends and potential future directions. This comprehensive view can facilitate the identification of knowledge gaps and areas for further investigation.

Of course, this study also has some shortcomings. Firstly, one limitation of this study is that it exclusively relies on data from the WoSCC database. This could potentially overlook relevant studies that are present in other databases. Secondly, another drawback is that the study focuses solely on publications written in English. This decision to filter out non-English papers may underestimate the overall impact of research published in other languages. Additionally, it is important to note that the study only accounted for literature available until August 1, 2023. This means that any papers published after this date would not be included in the analysis. Since the study aims to provide an overview of the entire year, there is a risk of not accurately representing the full extent of published literature for the year 2023.

The principle of VOSviewer analysis is mainly based on the construction and visualization of the relationship of network data (12). It can reflect the research topics of a discipline through high-frequency keywords and conduct cluster analysis using an algorithm based on the strength of associations. However, it has a single limitation on the position of elements and visualization methods, and cannot present research hotspots in a discipline (14, 79). Citespace mainly uses noun terms to detect research hotspots in disciplines. This algorithm easily identifies mutation words in the literature, making it more conducive for users to make reasonable predictions about the domain’s transitions, changes, and future development trends. The drawback is that it can visually display a certain amount of literature relationships, but the visualization effect is affected as the number of literature increases (13, 80). Bibliometrix is a bibliometric analysis tool based on the R language, which has powerful capabilities in literature analysis. However, it only supports the analysis of English literature and takes a long time to import R packages, affecting the overall analysis speed (11). Therefore, we combine the strengths and weaknesses of different analysis methods for a comprehensive analysis during the analysis process.

## Conclusions

Immunotherapy has shown significant research value and promising application prospects in the field of cervical carcinoma. This is evident from the increasing number of publications dedicated to studying immunotherapy in cervical carcinoma, indicating that scholars around the world are increasingly recognizing its potential. The United States and China are at the forefront of this research, leading the way in numerous studies. However, it is crucial to enhance collaboration and communication between countries and institutions to further expedite progress in this field.

High-risk HPV infection stands out as a notable factor contributing to the development of cervical cancer. Currently, vaccination serves as an essential tool in preventing cervical cancer by targeting the underlying infection. Nevertheless, there is still much to learn about the treatment of advanced cervical cancer. In comparison to traditional drugs, immunotherapeutic drugs offer significant advantages in the treatment of cervical carcinoma. Therefore, investigating and developing strategies for the use of immunotherapy in precision treatment will undoubtedly hold great application value in the future.

Furthermore, it is important to recognize that research efforts should extend beyond basic research. The translation of these findings to clinical applications, particularly in the use of immunotherapy for cervical carcinoma patients, deserves equal attention. By focusing on both fundamental research and practical implementation, we can maximize the potential impact of immunotherapy in the diagnosis and treatment of cervical carcinoma.

## Data availability statement

The original contributions presented in the study are included in the article/supplementary material. Further inquiries can be directed to the corresponding author.

## Author contributions

LS: Writing – original draft, Writing – review & editing. XL: Writing – original draft. MZ: Writing – original draft. QS: Writing – original draft. FL: Writing – review & editing, Writing – original draft.
